# Seroprevalence of *Toxoplasma gondii* infection among patients of a tertiary hospital in Guangzhou, Guangdong province, PR China

**DOI:** 10.1371/journal.pone.0286430

**Published:** 2023-07-10

**Authors:** Yu-bin Guan, Xiao-xiao Sun, Shao-lian Chen, Xiao-ting Zhu, Zhi-hua Zeng, Han-wei Lu, Hong-mei Feng, Yu Guo, Wen-gong Jiang, Kui Xiong, Xiao-rong Yang, Ho-Woo Nam, Zhao-shou Yang

**Affiliations:** 1 The First Affiliated Hospital/School of Clinical Medicine of Guangdong Pharmaceutical University, Guangdong Pharmaceutical University, Guangzhou, Guangdong, P.R. China; 2 Department of Parasitology, College of Medicine, Catholic University of Korea, Seoul, Republic of Korea; National Institute of Environmental Health Sciences National Toxicology Program Division, UNITED STATES

## Abstract

**Purpose:**

This study aimed to explore the prevalence of *Toxoplasma gondii* (*T*. *gondii*) among patients in Guangzhou city, South China, and to identify susceptible patient populations and analyze the causes of infection differences.

**Methods:**

From May 2020 to May 2022, a total of 637 sera were collected from patients, and 205 sera were collected from health participants as health control. All sera were examined by colloidal gold kits to detect the positivity of antibodies against *T*. *gondii*. And the positivity of antibodies in sera was confirmed with ARCHITECT i2000_SR_ system.

**Results:**

The prevalence of *T*. *gondii* infection in patients was 7.06% (45/637), which was lower than the prevalence in health participants 4.88% (10/205). Among patients, 34 (5.34%) were positive only for IgG, 10 (1.57%) were only for IgM, and 1 (0.16%) was positive for both IgG and IgM. There was a significant difference in prevalence between male and female patients, but not among different age groups or diseases groups. The prevalence of *T*. *gondii* infection in diseases groups varied. The prevalence was relatively high in patients with the disorders of thyroid gland and the malignant neoplasms of digestive organs, which suggests that caution should be taken to avoid *T*. *gondii* infection in these patients. Surprisingly, the prevalence was quite low in diffuse Large B-cell Lymphoma (DLBC) patients. This may be due to the overexpression of TNF-α in tumor tissues of DLBC patients and the higher protein level of TNF-α in sera of DLBC patients.

**Conclusion:**

This study provides a systematic exploration of the prevalence of *T*. *gondii* infection in patients in a tertiary hospital. Our data contributes to a better understanding of the epidemic investigation of *T*. *gondii* among patients in South China, which can help the prevention and treatment of the disease caused by *T*. *gondii* infection.

## Introduction

*Toxoplasma gondii* (*T*. *gondii*) is a globally infectious protozoan parasite, which is a food-borne pathogen that infects humans and animals [1]. *T*. *gondii* is usually transmitted by the oral route, organ transplantation and blood transfusion [2–5]. *T*. *gondii* infection causes toxoplasmosis. Although most patients with toxoplasmosis are asymptomatic, the infection can lead to serious diseases [6, 7]. *T*. *gondii* infection is strongly associated with retinochoroiditis, fatal toxoplasmic encephalitis and psychiatric disorders [7–10].

While numerous studies have been conducted to investigate the seroprevalence of *T*. *gondii* infection in China from different perspective of view [11–15], data on the prevalence of *T*. *gondii* among patients in South China is still insufficient. A study on ICU patients at the Third Affiliated Hospital of Sun Yat-sen University in Guangdong province, China was reported, but it lacked data from other departments of the hospital [16]. Song *et al*. analyzed parasite infection in patients who visit parasitological laboratory of Sun Yat-sen University, but they only collected sample from outpatients [17]. Xin *et al*. explored the prevalence of *T*. *gondii* infection using sera collected from the hospitals in the Guangdong province, China [18]. However, they did not categorize the enrolled populations by diseases type. Hence, it becomes essential to comprehensively investigate the prevalence of *T*. *gondii* infection among patients in South China. Guangzhou, the capital of Guangdong province in South China, has the largest population in South China [19, 20]. The first affiliated hospital of Guangdong Pharmaceutical University is a large tertiary general hospital located in Guangzhou, South China. Therefore, investigating the prevalence of infection in patients from this hospital could benefit for the understanding of the prevalence of *T*. *gondii* infection in South China.

*T*. *gondii* infection can be detected through direct and indirect methods. Direct methods, such as microscopic examination, polymerase chain reaction (PCR), bioassays to isolate parasites, are highly specific but often have low sensitivity. Indirect methods, such as serological detections of antibodies against antigens from *T*. *gondii*, are commonly used for the diagnosis of toxoplasmosis in animals and humans. However, these methods require skilled technicians to perform the operations [21, 22]. In our previous work, we improved a colloidal gold kit for the rapid detection of antibodies against *T*. *gondii* infection. The kit, named as rapid diagnostic test (RDT) kit, can simultaneously detect anti-*Toxoplasma* IgM and IgG antibodies using serum samples. The kit is fast and convenient to operate, and its sensitivity and specificity have been effectively verified [23]. Additionally, a follow-up epidemiological study of *T*. *gondii* infection in South Korea has been conducted using this kit [24].

In this study, we used RDT kits to investigate the prevalence of *T*. *gondii* infection among patients in Guangzhou, the capital of Guangdong province in South China. And we conducted a detailed analysis of the data and identified potential risk factors for *T*. *gondii* infection among different classification of diseases. Our findings contribute to the understanding of the epidemic investigation of *T*. *gondii* among patients in South China, which will help the prevention and treatment of the disease caused by *T*. *gondii* infection.

## Materials and methods

### Ethics statement

The study was approved by the Ethics Committee of the First Affiliated Hospital of Guangdong Pharmaceutical University (Approval Document No. [2020] (72)). Sample collection was performed in accordance with the relevant guidelines and regulations, and all participants read the relevant documents and agreed to participate in the study.

### Sample collection

We collected serum samples from patients at the First Affiliated Hospital of Guangdong Pharmaceutical University between May 2020 and May 2022 were collected. A total of 637 serum samples were randomly collected from patients, and an additional205 sera samples were collected from health participants. Fresh samples were tested to detect IgG or IgM antibodies against *T*. *gondii*. Samples that were not used on time were stored at -80°C until use. During the sample collection process, all participant information was kept anonymous and only age, health condition and gender were recorded.

### Rapid diagnostic test (RDT) and retest of positive samples

**Fig 1:**
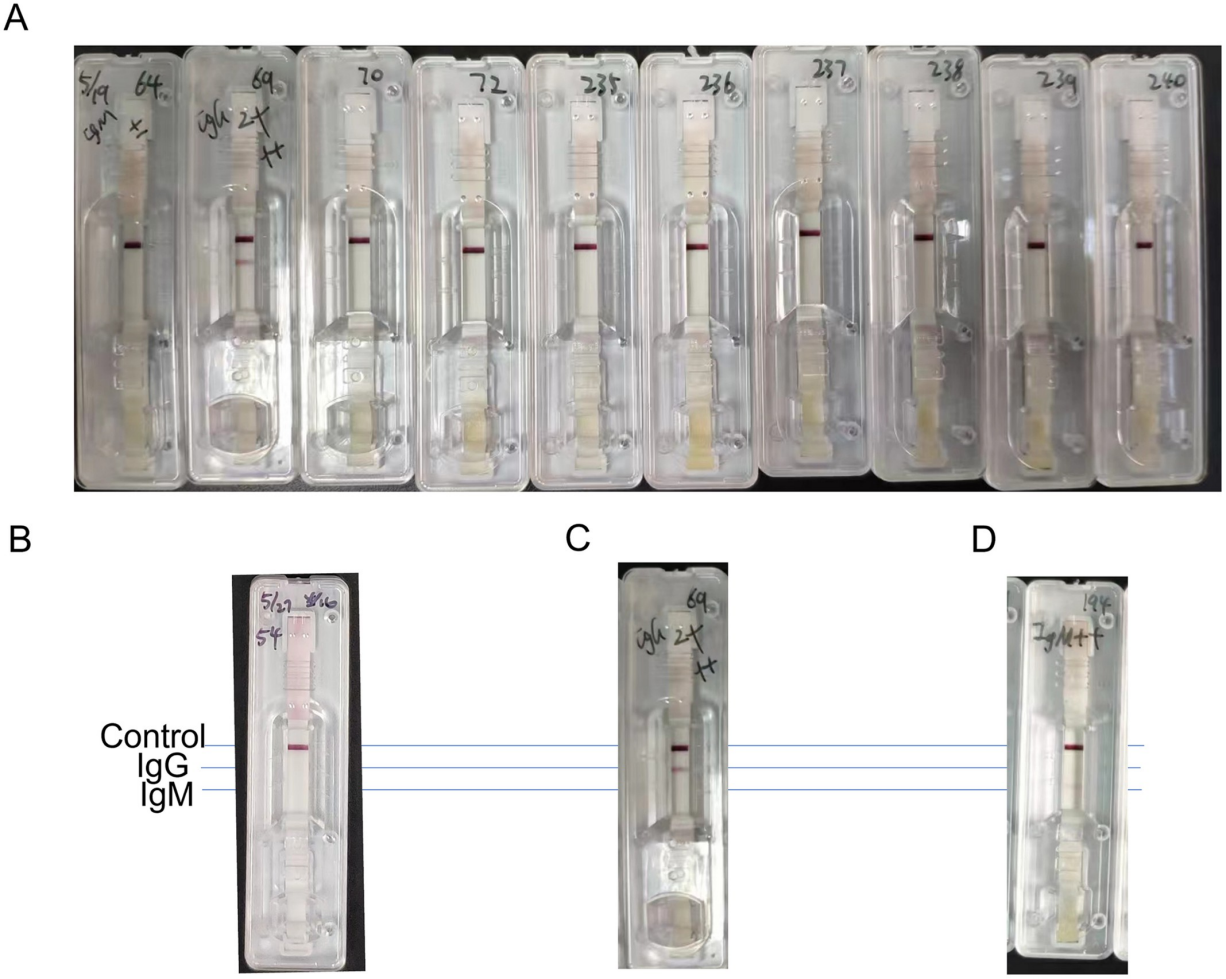
Colloidal gold kits used to detect IgG and IgM antibodies against *T*. *gondii*. (A) Modified colloidal gold kits (also known as Rapid diagnostic test (RDT) kits) used in this study. (B) The result was determined as “IgG-IgM-”. (C) The result was determined as “IgG+IgM-”. (D) The result was determined as “IgG-IgM+”. Note: “Control”, a band appears at this position, indicating that the detection is valid; “IgG”, a band appears at this position, indicating that there are IgG antibodies against *T*. *gondii* in samples; “IgM”, a band appears at this position, indicating that there are IgM antibodies against *T*. *gondii* in samples.

Modified colloidal gold kits (Genbody Inc., Cheonan, Korea), named as RDT kits [23, 24], were utilized to detect the presence of IgG or IgM antibodies against *T*. *gondii* in this study. The RDT kit contained recombinant protein (GST-GRA2-SAG1A) was applied in this RDT kit, and the operation procedure was in accordance with the manufacturer’s protocol. Positivity to IgG and IgM antibodies against *T*. *gondii* in sera were qualitatively detected [22]. Briefly, 10 μl of serum was added to the kit and eluted it with buffer. Reacting bands were judged after 10 minutes and the result was classified as “IgG-IgM-”, “IgG+IgM-”, “IgG-IgM+”and“IgG+IgM+”. A negative result was indicated by “IgG-IgM-”, a positive IgG but negative IgM antibody result was indicated by “IgG+IgM-”, a positive IgM but negative IgG antibody result was indicated by “IgG-IgM+”, and a positive result for both IgG and IgM antibodies was indicated by “IgG+IgM+” (as shown in Fig 1). Three investigators reviewed the results, and any samples that were positive for either IgG or IgM against *T*. *gondii* were retested and confirmed with the ARCHITECT i2000SR system (Abbott, Abbott Park, Illinois, U.S.A.) in accordance with the Clinical and Laboratory Standards Institute (CLSI) [25].

### GEPIA database analysis

The Gene expression profiling interactive analysis (GEPIA) database (http://gepia.cancer-pku.cn/) is a web server for gene expression profiling and interaction analysis for cancer and normal tissues [26]. The database presents gene expression profiles across all normal tissues and tumor samples in GEPIA, allowing for comparative analysis and interactive exploration.

### Cytokines test

The ELISA kits for detecting TNF-α and IL-10 were purchased from Jiyinmei bio-company (Wuhan, China). The protein levels of TNF-α and IL-10 in the sera were determined using the ELISA kits following the manufacturer’s instructions.

### Statistical analysis

The test results are classified as either negative or positive. Pearson Chi-Square and Fisher’s exact tests were used to investigate the differences of the prevalence between patients from different aspects. Statistical analysis was performed using SPSS software (SPSS Inc., Chicago, Illinois) and R (v.4.0.5). A *P* value below 0.05 was considered significant.

## Results

### Prevalence of *T*. *gondii* in patients in Guangzhou, South China

**Fig 2:**
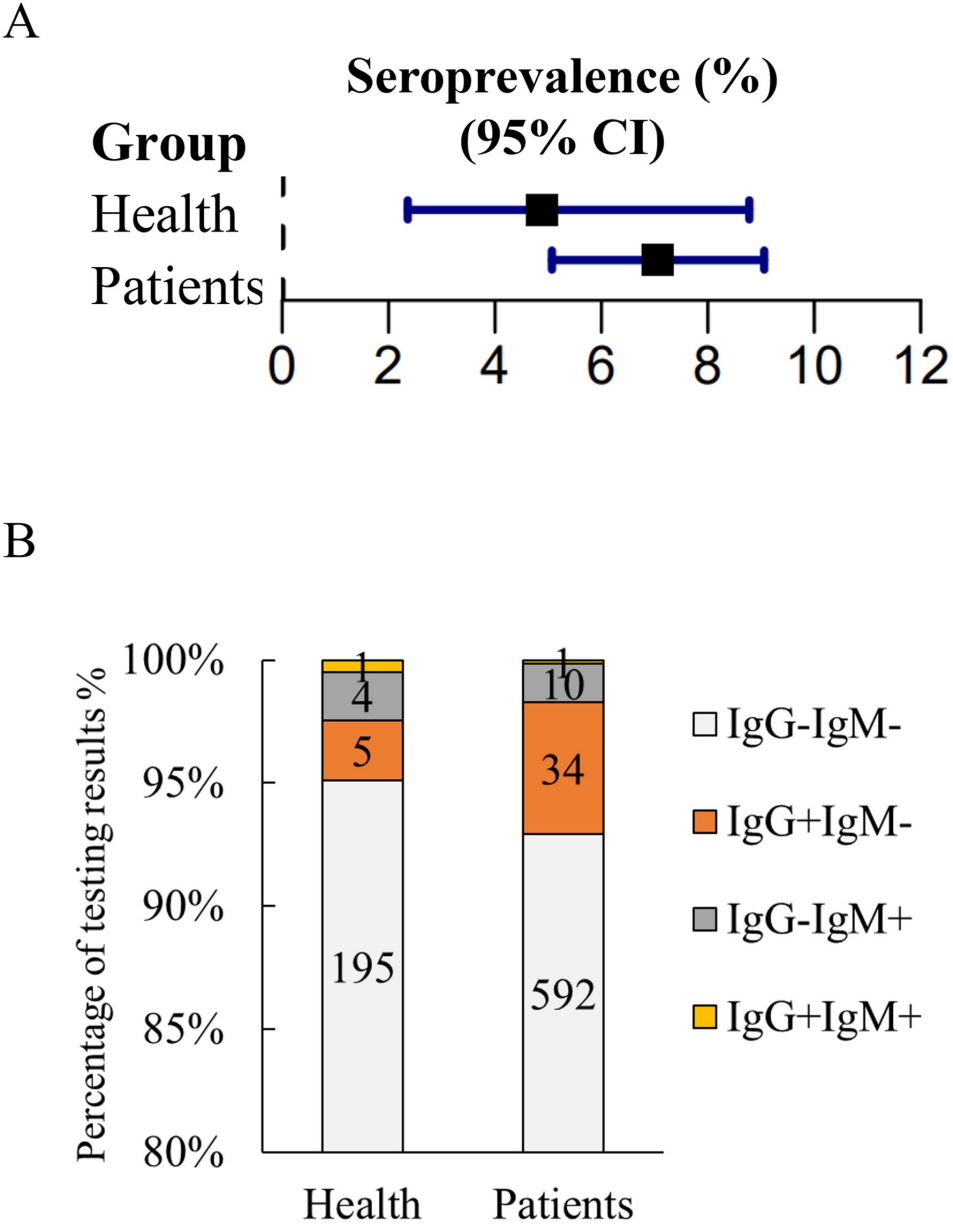
Seroprevalence of *T*. *gondii* infection. (A) Comparison of the seroprevalence of *T*. *gondii* among health individuals and patients, χ^2^ = 1.554, *P* = 0.213. (B) The bar charts show the percentage of test results. The numbers in the light grey boxes in the bar charts represent the number of patients who are tested negative for both IgG and IgM antibody, i.e., “IgG-IgM-”. The numbers in the orange-red boxes in the bar charts represent the number of patients who are tested positive for IgG but not IgM antibody, i.e., “IgG+IgM-”. The numbers in the dark grey boxes in the bar charts represent the number of patients who are tested positive for IgM but not IgG antibody, i.e., “IgG-IgM+”. The numbers in the orange boxes in the bar charts represent the number of patients who are tested positive for both IgM and IgG antibodies, i.e., “IgG+IgM+”.

**Fig 3:**
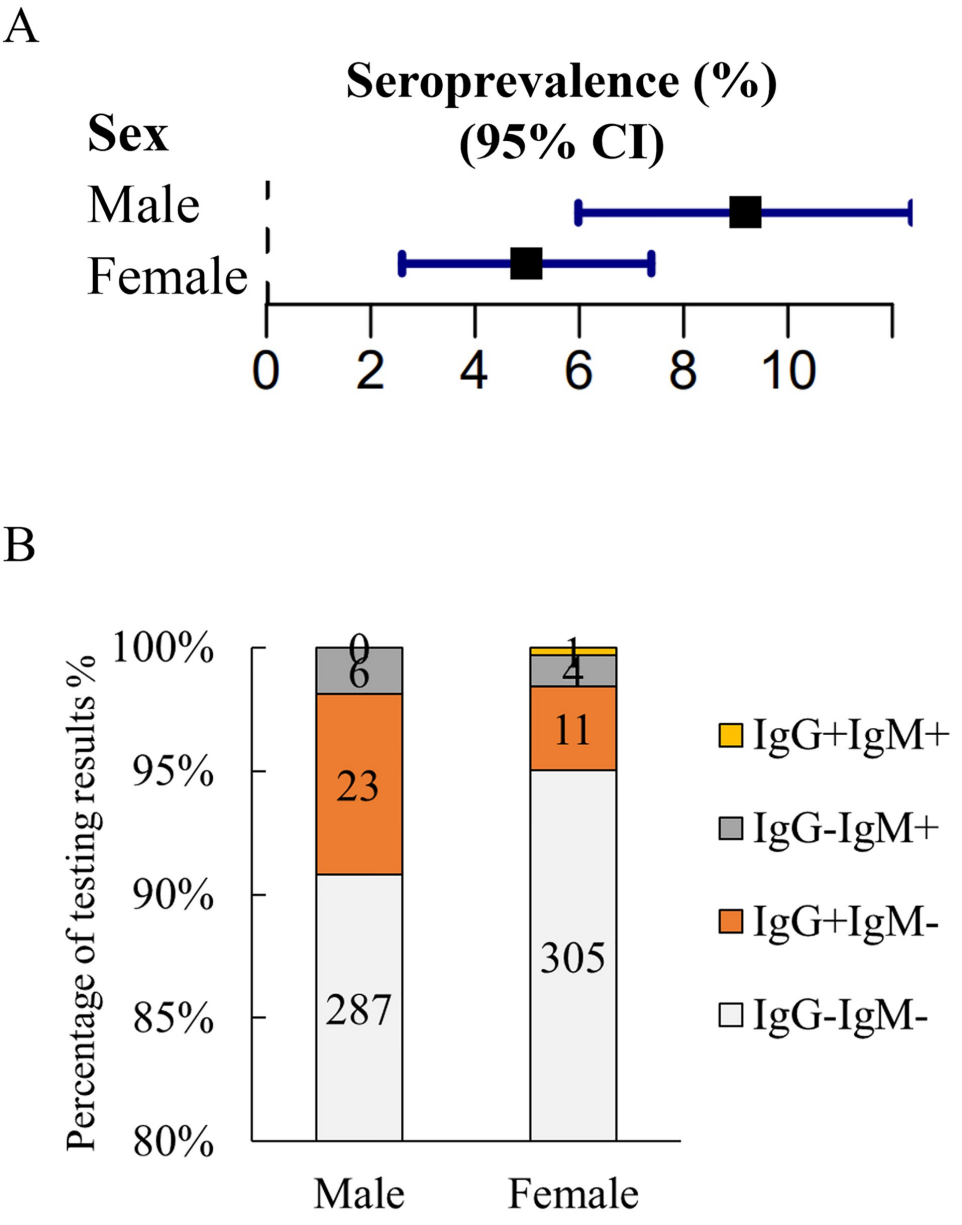
Seroprevalence of *T*. *gondii* in patients by sex. (A) Comparison of seroprevalence of *T*. *gondii* infection between male and female patients, χ^2^ = 3.982, *P = 0.046. (B) The percentage of testing results in different sex groups.

**Fig 4:**
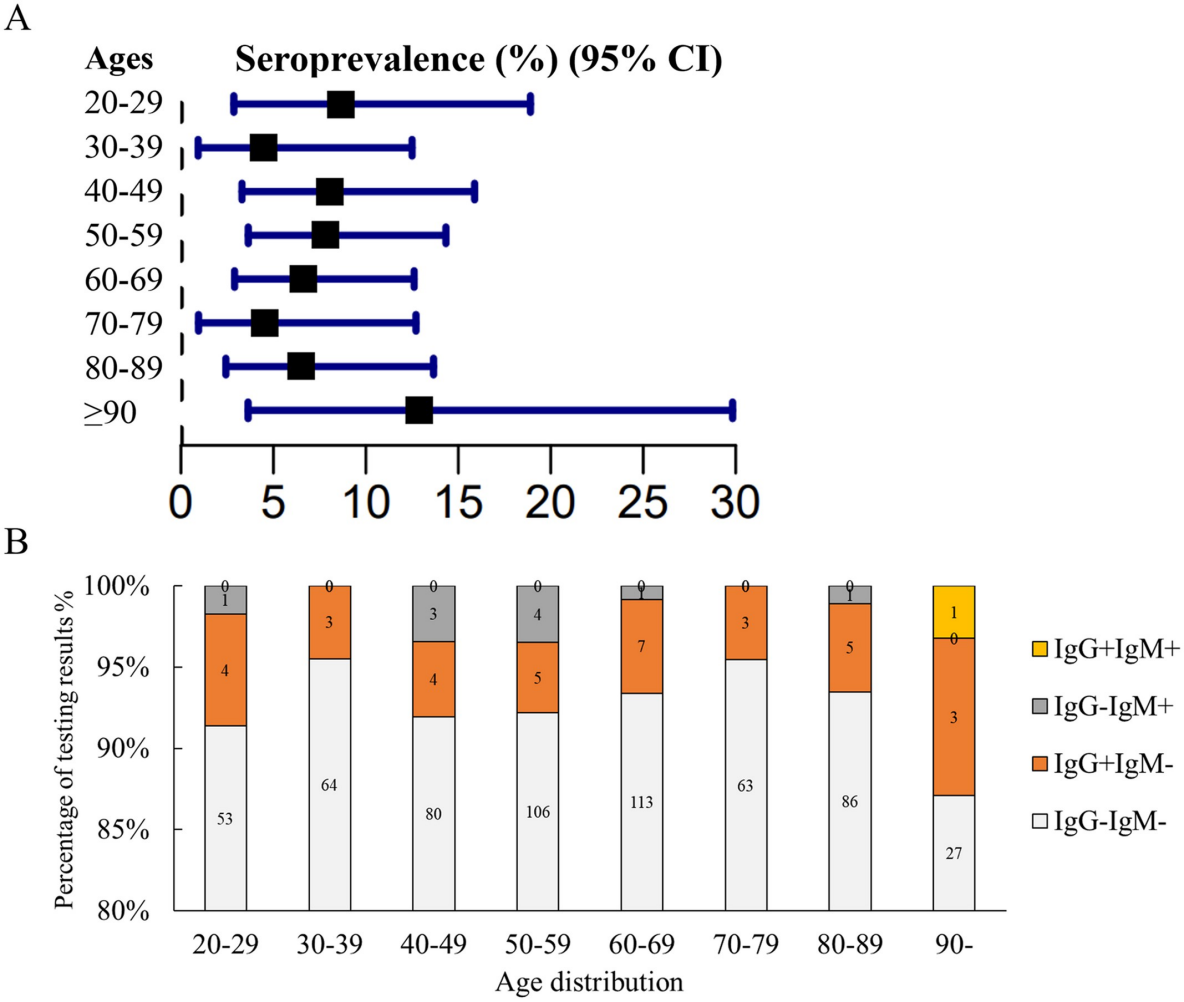
Seroprevalence of *T*. *gondii* infection in patients by age. (A) Comparison of seroprevalence of *T*. *gondii* among patients in different age groups, χ^2^ = 3.833, *P* = 0.802. (B) The percentage of testing results in different age groups.

To investigate the prevalence of *T*. *gondii* among individuals in Guangzhou, South China, we collected serum samples from 205 healthy people and 637 patients. Out of the 882 serum samples,55 samples were positive for anti-*T*. *gondii* antibodies using RDT kit, and these positive test results were subsequently confirmed with ARCHITECT i2000SR system. The overall seroprevalence of *T*. *gondii* infection in the healthy participants was 4.88% (10/205). Although the difference in the prevalence between healthy individuals and patients is not statistically significant, the prevalence was higher in patient group (Table 1 and Fig 2). The overall seroprevalence of *T*. *gondii* infection among patients was of 7.06%, with 45 out of 637 patients testing positive for IgG or IgM antibodies against *T*. *gondii* (Table 1 and Fig 2). Among the positive patients, 34 (5.33%) were seropositive for only IgG, 10 (1.57%) for only IgM, and 1 (0.16%) for both IgG and IgM (Fig 2B). The prevalence of *T*. *gondii* infection in patients varied significantly by sex, but not by age group (Table 2, and Figs 3 & 4).

**Table 1:** Seroprevalence of *T*. *gondii* infection across all participants in this study.

Characteristics	No. of each type of test results (n)				No. of positive/ examined	Seroprevalence (%) (95% CI)	Statistical analysis
	IgG-IgM-	IgG+IgM-	IgG- IgM+	IgG+IgM+			
Health	195	5	4	1	10/205	4.88(2.36–8.78)^a^	*χ*^*2*^ = 1.214, *P* = 0.271^c^
Patients	592	34	10	1	45/637	7.06(5.07–9.06)^b^	

^a^, Confidence intervals are calculated by the Clopper-Pearson Method;

^b^, Confidence intervals are calculated by the normal approximation method;

^c^, Pearson’s chi-squared test was applied to do statistical analysis.

**Table 2:** Seroprevalence of *T*. *gondii* infection in patients by sex and age.

Characteristics	No. of each type of test results (n)				No. of positive/ examined	Seroprevalence (%) (95% CI)	Statistical analysis
	IgG-IgM-	IgG+IgM-	IgG- IgM+	IgG+IgM+			
Sex							
Male	287	23	6	0	29/316	9.18(5.98–12.38)^a^	*χ*^*2*^ = 4.264, *P* = 0.039 ^c^
Female	305	11	4	1	16/321	4.98(2.60–7.38) ^a^	
Age (Years)							
20–29	53	4	1	0	5/58	8.62(2.86–18.9) ^b^	*χ*^*2*^ = 3.550, *P* = 0.835 ^d^
30–39	64	3	0	0	3/67	4.48(0.93–12.5) ^b^	
40–49	80	4	3	0	7/87	8.05(3.30–15.88) ^b^	
50–59	106	5	4	0	9/115	7.82(3.64–14.33) ^b^	
60–69	113	7	1	0	8/121	6.61(2.90–12.61) ^b^	
70–79	63	3	0	0	3/66	4.55(0.95–12.71) ^b^	
80–89	86	5	1	0	6/92	6.52(2.43–13.66) ^b^	
≥90	27	3	0	1	4/31	12.90(3.63–29.83) ^b^	

^a^, Confidence intervals are calculated by the normal approximation method;

^b^, Confidence intervals are calculated by the Clopper-Pearson Method;

^c^, Pearson’s chi-squared test was applied to do statistical analysis;

^d^, fisher exact test was applied to do statistical analysis.

### Prevalence of *T*. *gondii* infection among patients with different types of diseases

**Fig 5:**
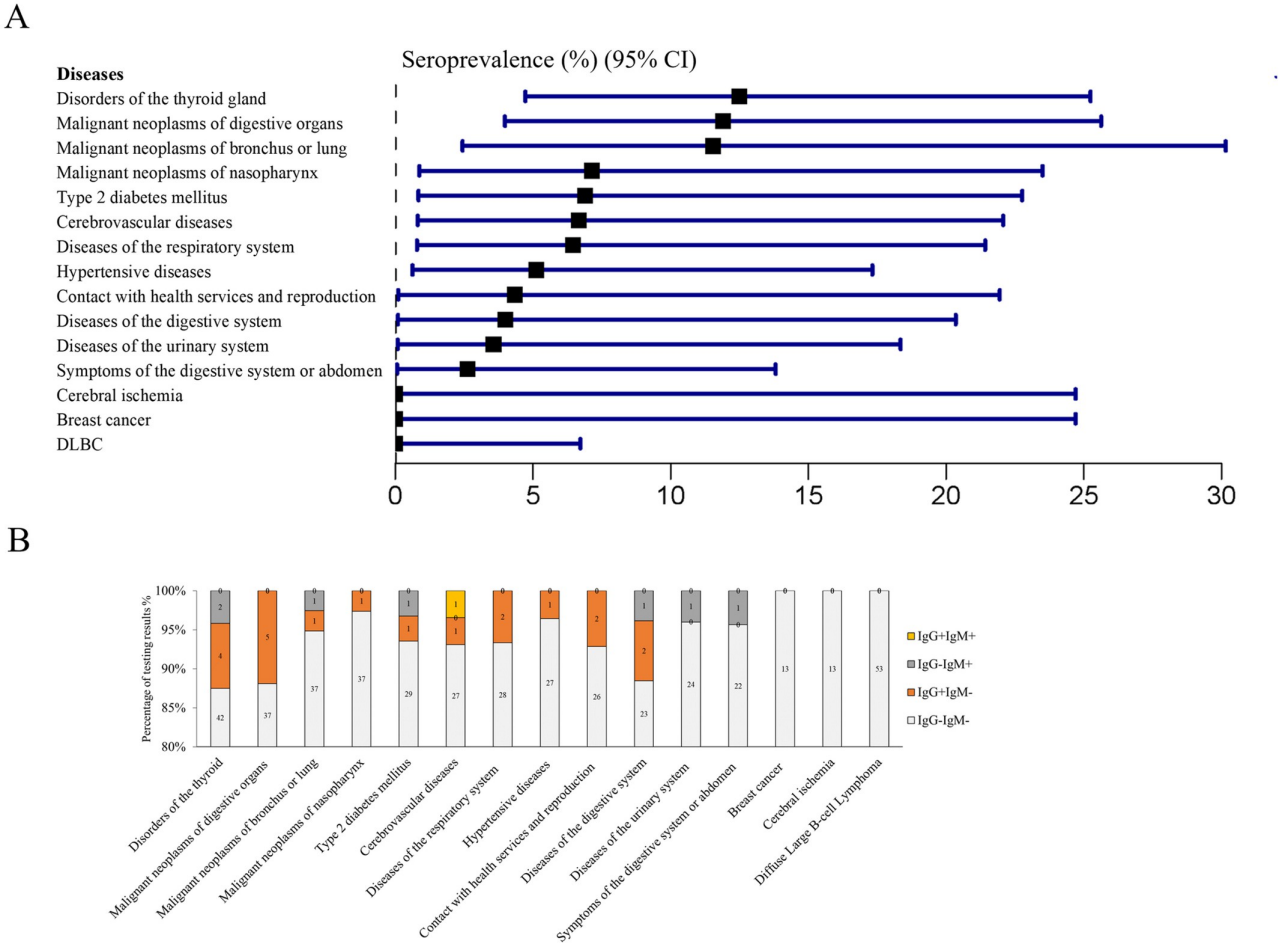
Seroprevalence of *T*. *gondii* infection based on the classification of diseases in this study. (A) The overall seroprevalence of *T*. *gondii* infection in different types of diseases, with a Fisher exact test revealing no significant difference (*P*>0.05). (B) The percentage of testing results in different types of disease groups.

Fig 3 showed that sex could be one of the potential risk factors for *T*. *gondii* among patients. Then we wonder whether the types of the diseases are the risk factor. The diagnosis of diseases for patients was based on International Classification of Diseases (ICD 11) (http://www.who.int/news-room/detail/18-06-2018-who-releases-newinternational-classification-of-diseases-icd-11). We further categorized similar diseases into one group based on the code from ICD 11 to minimize the number of disease groups. To ensure the validity of the analysis, we selected a group with a sample size greater than twelve to analyze the differences between groups. Stratification by diseases did not show a difference in prevalence between groups (Table 3 and Fig 5). As results, patients diagnosed with disorders of the thyroid gland, malignant neoplasms of digestive organs, and diseases of the digestive system had a considerably high prevalence of antibodies to *T*. *gondii*. By contrast, the prevalence was very low in patients diagnosed with diffuse Large B-cell Lymphoma (DLBC), cerebral ischemia, and malignant neoplasms of breast (breast cancer).

**Table 3:** Seroprevalence of *T*. *gondii* infection in different types of diseases.

Characteristics	No. of each type of test results (n)				No. of positive/ examined	Seroprevalence (%) (95% CI)^a^	Statistical analysis^b^
	IgG-IgM-	IgG+ IgM-	IgG- IgM+	IgG+IgM+			
Disorders of the thyroid gland	42	4	2	0	6/48	12.50(4.73–25.25)	*P* = 0.418
Malignant neoplasms of digestive organs	37	5	0	0	5/42	11.90(3.98–25.63)	
Malignant neoplasms of bronchus or lung	37	1	1	0	2/39	5.13(0.63–17.32)	
Malignant neoplasms of nasopharynx	37	1	0	0	1/38	2.63(0.07–13.81)	
Type 2 diabetes mellitus	29	1	1	0	2/31	6.45(0.79–21.42)	
Cerebrovascular diseases	27	1	0	1	2/29	6.90(0.85–22.77)	
Diseases of the respiratory system	28	2	0	0	2/30	6.67(0.82–22.07)	
Hypertensive diseases	27	1	0	0	1/28	3.57(0.09–18.35)	
Contact with health services and reproduction	26	2	0	0	2/28	7.14(0.88–23.50)	
Diseases of the digestive system	23	2	1	0	3/26	11.54(2.45–30.15)	
Diseases of the urinary system	24	0	1	0	1/25	4.00(0.10–20.35)	
Symptoms of the digestive system or abdomen	22	0	1	0	1/23	4.35(0.11–21.95)	
Cerebral ischemia	13	0	0	0	0/13	0.00(0.00–24.71)	
Breast cancer	13	0	0	0	0/13	0.00(0.00–24.71)	
DLBC	53	0	0	0	0/53	0.00(0.00–6.72)	

^a^, Confidence intervals are calculated by the Clopper-Pearson Method;

^b^, Monte Carlo method was applied to do statistical analysis.

### *T*. *gondii* invasion related cytokines expression in cancer patients

**Fig 6:**
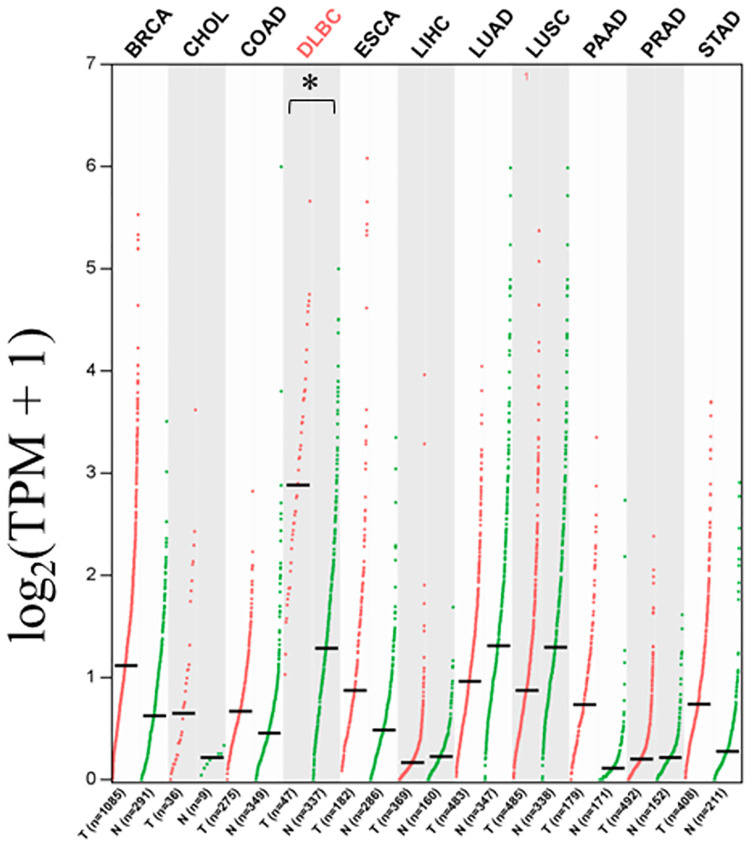
The expression profile of TNF-α gene expression across tumor samples and normal tissues in GEPIA. TPM (Transcripts Per Million) values are indicated., Transcripts Per Million. The asterisk (*) indicates statistical significance at *P*< 0.05. We analyzed five types of malignant neoplasms of digestive organs (Cholangiocarcinoma (CHOL), Colon adenocarcinoma (COAD), Liver hepatocellular carcinoma (LIHC), Pancreatic adenocarcinoma (PAAD), Rectum adenocarcinoma (READ), Stomach adenocarcinoma (STAD)), two types of malignant neoplasms of bronchus or lung (Lung squamous cell carcinoma (LUSC), Lung adenocarcinoma (LUAD)), one type of malignant neoplasms of nasopharynx (Esophageal carcinoma (ESCA)), one type of lymphoma (Lymphoid Neoplasm Diffuse Large B-cell Lymphoma (DLBC)), and one type of breast cancer (Breast invasive carcinoma (BRCA)).

**Fig 7:**
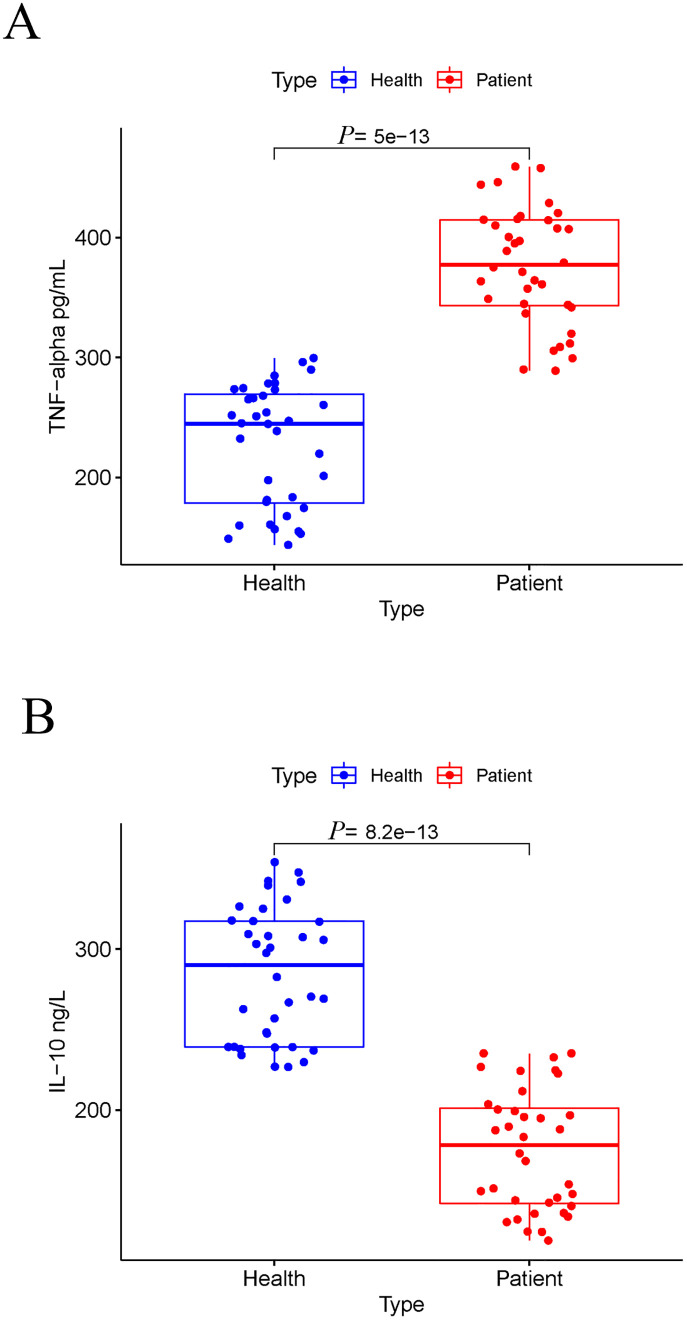
The protein levels of TNF-α and IL-10 in the sera of DLBC patients and health individuals. The cytokine levels were measured using a commercial ELISA kit, and the results were presented in box plots for comparison. The protein levels of TNF-α (A) and IL-10 (B) were significantly different between the DLBC patients and age-gender-matched healthy individuals.

In our study, we investigated the prevalence of *T*. *gondii* infection in patients with various types of neoplasms, including malignant neoplasms of digestive organs, bronchus or lung, nasopharynx, lymphoma, and breast cancer. A total of 217 neoplasms patients were enrolled in this study, and the prevalence was quite various among these patients. To explore the association between anti-*T*. *gondii* genes expression and the type of neoplasms, we utilized a free public cancer database. Tumor necrosis factor-alpha (TNF-α) plays a crucial role in host immunity against *T*. *gondii* infection [27, 28]. Therefore, we analyzed the expression of TNF-α in the malignant neoplasms of digestive organs, bronchus or lung, nasopharynx, DLBC and breast cancer from GEPIA. The bioinformatic analysis showed that TNF-α was significantly more highly expressed in DLBC tumors samples than in other tumors and normal tissues (Fig 6). Interestingly, the prevalence of *T*. *gondii* infection in DLBC patients enrolled in this study was also very low (0/53). We further tested the protein level of TNF-α in the sera of 36 DLBC patients and 36 age-and gender-matched healthy individuals. The result showed that the protein level of TNF-α in DLBC patients’ sera was significantly higher than that in health individuals (Fig 7A). We also tested the protein level of IL-10 in sera, which can enhance *T*. *gondii* invasion into cells at the initial stage of the infection [29]. As expected, the IL-10 in DLBC patients’ sera was significantly lower than that in health individuals (Fig 7B).

## Discussion

Although previous studies have investigated *T*. *gondii* infection among patients in South China has been investigated [16, 17], the data on the seroprevalence of *T*. *gondii* in South China is still insufficient. Here, we studied the prevalence of *T*. *gondii* infection among patients in Guangzhou, an important city in South China. The overall prevalence *T*. *gondii* infection in patients was 6.65% in this study, which is close to the average prevalence previously reported in the general Chinese population, lower than the global average [18, 30]. This may be attributed to the unique dietary and cooking habits in China [31].

This study focused on the prevalence of *T*. *gondii* infection among patients. The results of prevalence were deeply analyzed. It was noteworthy that the prevalence of *T*. *gondii* infection in male was significantly higher than that in female in this study, consistent with our previous reports from another East Aisa country, South Korea [22, 24], and a recent report from Serbia [32]. This indicates that sex could be one of the potential risk factors in South China. Studies from other region of the world also showed the similar phenomena [33, 34], except some opposite results due to regional and cultural habits [35]. The difference in infection rates between male and female is speculated to be related to sex hormones [36]. For example, prolactin, which is lower in males than females [37], has been reported to have strong anti-*T*. *gondii* effects [38]. Progesterone, which is several-fold higher in pregnant females, induces the egress of intracellular *T*. *gondii* [39]. While intracellular *T*. *gondii* could migrate through the blood-brain barrier (BBB) in a ’Trojan horse’ manner to from chronic infection [40, 41], early egressed *T*. *gondii* might failed to directly go through the BBB in this way. At the initial stage of infection, extracellular *T*. *gondii* could be fast eliminated by the host’s innate immunity [28]. Without sustained antigen stimulation, the antibody titer in the host will be reduced or even undetectable [42]. Therefore, it is assumed that at the initial stage of *T*. *gondii* infection female sex related hormones such as prolactin and progesterone induce the egress of intracellular infected *T*. *gondii*, which will be further eliminated by host innate immune cells, such as neutrophil. Eliminated *T*. *gondii* could not migrate into immune-exempt organs such as the brain to from chronic infection. This may explain the difference of prevalence of antibodies against *T*. *gondii* among sex.

In the present study, though we did not find a significant difference in infection rates among diseases (as shown in Fig 5). However, we observed a higher prevalence in patients with three types of diseases, including disorders of the thyroid gland, malignant neoplasms of digestive organs and diseases of the digestive system, which should be well cared to avoid *T*. *gondii* infection. The high prevalence in disorders of the thyroid gland was consistent with some reports that *T*. *gondii* infection might be positively associated with thyroid disease [43]. But some studies have given the opposite result, which requires further research [43]. The high prevalence in malignant neoplasms of digestive organs were also reported by other Chinese research teams [44, 45]. And the high prevalence in disease of digestive system or organs perhaps result from the impaired mucosal defenses [44, 45].

In addition, we found a very low prevalence of *T*. *gondii* infection in patients with DLBC lymphoma (Fig 5), suggesting a possible negative association between this disease and *T*. *gondii* infection. Anemia, which is commonly associated with of lymphoma progression, can make patients suspectable to the infections [46–48]. In this study, all enrolled lymphoma patients did not suffer from anemia (data not published), this may explain the low prevalence in this group of patients. Another main reason for the low prevalence of *T*. *gondii* infection in DLBC lymphoma patients is the differential expression of cytokines in cancer tissues and sera. Pretreatment with TNF-α could protect mice from *T*. *gondii* infection [27]. While another cytokine IL-10 promotes *T*. *gondii* invasion into the cells [29]. Our bioinformatic analysis showed that RNA expression of TNF-α is over-expressed in tumor tissues of DLBC patients (as shown in Fig 6). Further analysis in this study found that the protein level of TNF-α in DLBC patients’ sera is significantly higher than that in health individuals, while the protein level of IL-10 in DLBC patients’ sera is significantly lower (as shown in Fig 7). Hence high expression of TNF-α and low expression of IL-10 may result in the low infection rates in this patient population.

Another finding in this study is that the prevalence in breast cancer patients is very low compared to other type of cancers, which is consistent with the results from other research groups [45]. This may be attributed to the fact that prolactin, a hormone involved in the development of breast cancer, also exhibits potential anti-Toxoplasma gondii effects, as previously mentioned [38, 49, 50].

In summary, this study provides a systematic assessment of the prevalence of *T*. *gondii* infection among patients in a tertiary hospital located in Guangzhou, an important city in South China. Our findings identify specific at-risk populations that would benefit from avoidance of toxoplasmosis infection sources of. This data contributes to the understanding of *T*. *gondii* prevalence among patients in South China, and may ultimately aid in the prevention and treatment of diseases caused by this pathogen.
